# Disassembly of Tau fibrils by the human Hsp70 disaggregation machinery generates small seeding-competent species

**DOI:** 10.1074/jbc.RA120.013478

**Published:** 2020-05-28

**Authors:** Eliana Nachman, Anne S. Wentink, Karine Madiona, Luc Bousset, Taxiarchis Katsinelos, Kieren Allinson, Harm Kampinga, William A. McEwan, Thomas R. Jahn, Ronald Melki, Axel Mogk, Bernd Bukau, Carmen Nussbaum-Krammer

**Affiliations:** 1 Center for Molecular Biology of Heidelberg University (ZMBH) and German Cancer Research Center (DKFZ), DKFZ-ZMBH Alliance, Heidelberg, Germany; 2 Schaller Research Group Proteostasis in Neurodegenerative Disease of Heidelberg University and German Cancer Research Center (DKFZ), Heidelberg, Germany; 3 Institute Francois Jacob (MIRCen), CEA, and Laboratory of Neurodegenerative Diseases, CNRS, Fontenay-Aux-Roses, France; 4 Department of Clinical Neurosciences, UK Dementia Research Institute at the University of Cambridge, Cambridge, United Kingdom; 5 Department of Neuropathology, Cambridge Universities Hospital Trust, Cambridge, United Kingdom; 6 Department of Biomedical Science of Cell and System, University Medical Center Groningen, University of Groningen, Groningen, The Netherlands

**Keywords:** Tau protein, tauopathy, proteostasis, amyloid, protein aggregation, neurodegenerative disease, 70-kilodalton heat shock protein (Hsp70), chaperone DNAJ (DNAJ), molecular chaperone, prion, Tau, chaperone DnaJ (DnaJ)

## Abstract

The accumulation of amyloid Tau aggregates is implicated in Alzheimer's disease (AD) and other tauopathies. Molecular chaperones are known to maintain protein homeostasis. Here, we show that an ATP-dependent human chaperone system disassembles Tau fibrils *in vitro*. We found that this function is mediated by the core chaperone HSC70, assisted by specific cochaperones, in particular class B J-domain proteins and a heat shock protein 110 (Hsp110)-type nucleotide exchange factor (NEF). The Hsp70 disaggregation machinery processed recombinant fibrils assembled from all six Tau isoforms as well as Sarkosyl-resistant Tau aggregates extracted from cell cultures and human AD brain tissues, demonstrating the ability of the Hsp70 machinery to recognize a broad range of Tau aggregates. However, the chaperone activity released monomeric and small oligomeric Tau species, which induced the aggregation of self-propagating Tau conformers in a Tau cell culture model. We conclude that the activity of the Hsp70 disaggregation machinery is a double-edged sword, as it eliminates Tau amyloids at the cost of generating new seeds.

Amyloid deposits are characteristic of various neurodegenerative diseases, such as Alzheimer's disease (AD) and Parkinson's disease (PD). Typically, symptoms surface only at advanced age, indicating that a buffering system exists that prevents disease onset and amyloid formation earlier in life (1).

Disease-associated proteins aggregate into amyloid fibrils characterized by their highly ordered β-sheet structure (2). The monomeric form of these proteins populates conformations susceptible to aggregation, leading to the formation of a variety of assemblies of various molecular weights. Some have seeding propensities that trigger further aggregation into fibrils by templated incorporation of the monomeric form of the constituting protein in a conformation that is compatible with the fibril ends. This templated propagation of the amyloid structure is thought to be the basis for the prion-like spreading of pathological inclusions and toxicity in neurodegenerative diseases (3).

The aggregation of the microtubule-associated protein Tau is implicated in ∼20 different diseases, termed tauopathies, with AD being the most common form of dementia (4). Thus, Tau is the most frequently aggregating protein in human neurodegenerative diseases. Under physiological conditions, Tau is highly soluble and expressed as six alternatively spliced isoforms (5). It binds and supports the assembly of microtubules that are vital for axonal transport in neurons (6). Under pathological conditions, the affinity of Tau to microtubules is reduced, either by disease-associated mutations or hyperphosphorylation (7–9). Detached Tau then forms aggregates in the cytoplasm that eventually evolve into fibrillar inclusions in affected neurons or glial cells (4).

In healthy cells, the homeostasis of Tau and other proteins is tightly controlled by a protein quality control network, including molecular chaperones (1, 10, 11). This quality control system protects the proteome by regulating the synthesis, folding, and trafficking of native proteins to their subcellular destination as well as the refolding and degradation of misfolded species. As such, molecular chaperones act at every step of the amyloid formation and clearance process (10, 12–15).

Numerous studies have linked molecular chaperone action to Tau aggregation both *in vitro* and *in vivo*. It was shown that individual heat shock protein of 70 kDa (Hsp70) family members, several J-domain protein cochaperones, HSP60, and the small heat shock protein HSP27 delay Tau fibril formation (16–19). Moreover, Hsp70 chaperones interact with oligomeric Tau and prevent further aggregation into fibrils (18). So far, only inefficient disassembly of preformed Tau fibrils by Hsp70 activity has been observed (18, 19). Our work and other studies demonstrated that Hsp70 disaggregation activity strongly relies on a coordinated action of an Hsp70 core chaperone with specific cochaperones (13, 15). As the effects of chaperones and cochaperones on Tau amyloid fibrils were usually examined individually and not in combination, only the prevention of aggregation and a slight shift of the amyloid equilibrium toward the soluble fraction because of their binding to intermediates was observed but no specific disaggregation activity of preformed Tau fibrils. Furthermore, a tightly timed on-off switching of chaperone and substrate expression would be necessary to distinguish between the prevention of aggregation and disaggregation activity by chaperones in *in vivo* models, which is usually not done. J-domain proteins deliver clients to Hsp70 by preselecting them and activating their ATP hydrolysis-dependent binding into the substrate binding pocket of Hsp70, thereby determining substrate specificity of the machinery. Nucleotide exchange factors (NEFs) regulate the lifetime of the Hsp70-substrate complexes, which determines substrate fate, such as refolding, transfer to other chaperone systems, or handover to the degradation machinery (20). To date, the correct composition of chaperones and cochaperone combinations that efficiently dissolves Tau fibrils is unknown.

Here, we demonstrate that the human Hsp70 disaggregation machinery, referred to here as Hsp70 disaggregase, can disassemble amyloid Tau fibrils *in vitro*. The Hsp70 disaggregase is an ATP-dependent chaperone system that is comprised of the constitutively expressed Hsp70 family member HSPA8 (HSC70), the J-domain proteins DNAJB1 or DNAJB4, and HSPA4 (APG2), an Hsp110-type NEF. Recombinant fibrils of all six Tau isoforms, as well as Sarkosyl-resistant Tau aggregates extracted from a cell culture model or AD brain tissue, could be processed by this chaperone system, demonstrating that this chaperone machinery can disintegrate disease-relevant amyloids. We further show that class B J-domain proteins are essential for this activity and that there is partial redundancy within this class of chaperones, whereas class A J-domain proteins were not able to support Hsp70 disaggregase function. The disaggregation reaction produced monomeric Tau as well as small oligomers. Importantly, Tau species liberated by the disaggregation reaction were seeding competent and induced the formation of Tau foci in a biosensor cell line for Tau aggregation, which were stably inherited over multiple cell passages.

This study shows that Hsp70 disaggregase activity can be extended to the most prevalent neurodegenerative diseases involving Tau. Although almost completely depolymerized to monomers, the fraction of Tau that was released from amyloid fibrils by chaperone action was still seeding competent. As the generation of seeding-competent species might boost the prion-like propagation of amyloid Tau aggregates, it needs to be examined whether chaperone-mediated Tau disaggregation may exacerbate the associated neurotoxicity *in vivo*.

## Results

### The human Hsp70 disaggregase disassembles recombinant Tau fibrils in vitro

**Figure 1.**
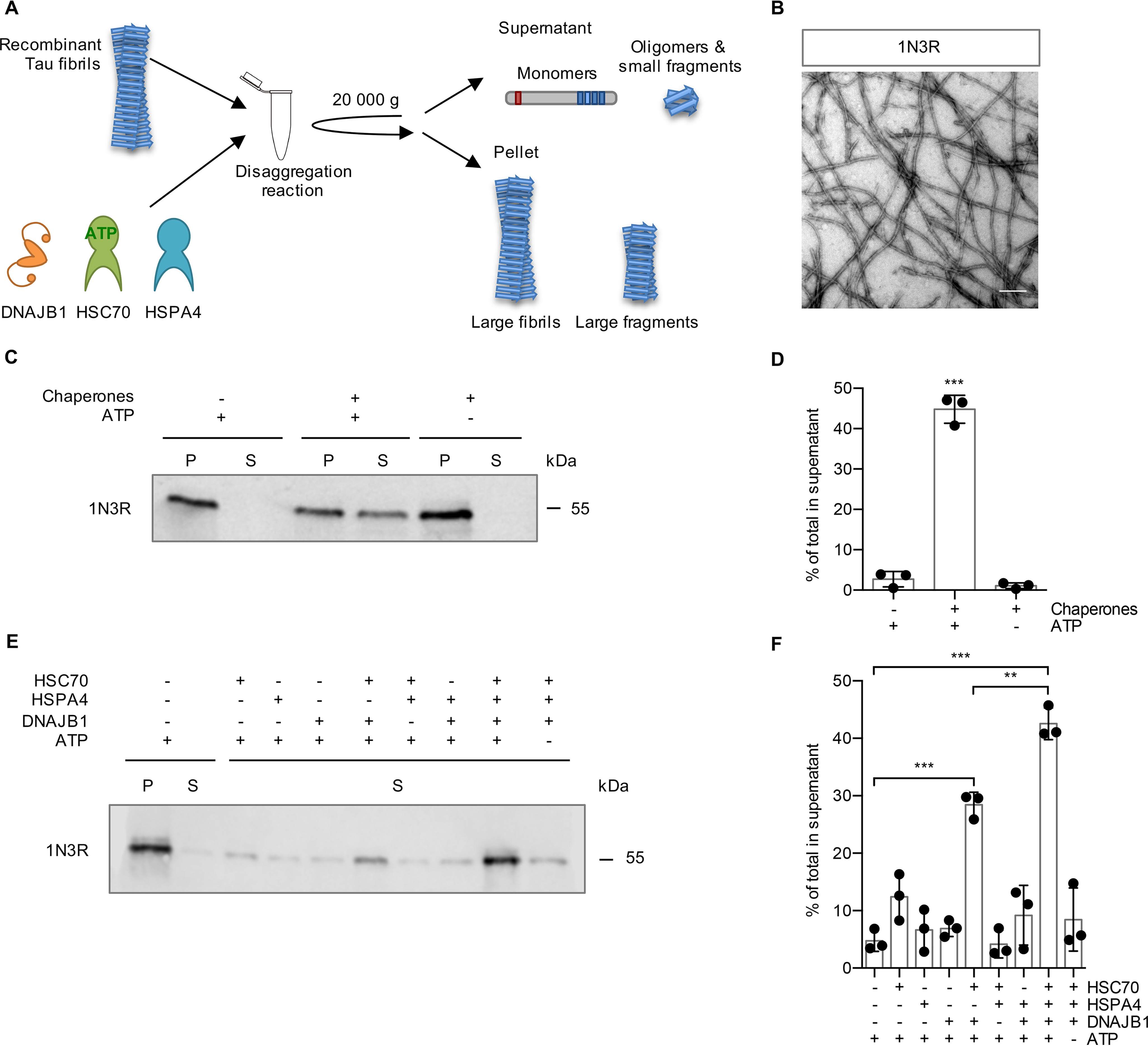
**The human Hsp70 disaggregation machinery disassembles recombinant Tau fibrils.** *A*, experimental setup of the disaggregation reaction and subsequent sedimentation assay. Tau fibrils were treated with chaperones for 20 h at 30 °C. Large fibrils are separated from smaller species and monomers by centrifugation at 20,000 × *g*. *B*, electron micrograph of negatively stained fibrils from recombinant 1N3R Tau assembled *in vitro*. *Scale bar*, 250 nm. *C*, fibrils (2 μm) were incubated with chaperones (4 μm HSC70, 2 μm DNAJB1, 0.2 μm HSPA4) ± ATP for 20 h at 30 °C. S and P fractions were separated by centrifugation (20,000 × *g*) and analyzed by immunoblotting. *D*, densitometric quantification of Tau in S fractions compared with the total (S + P) of each sample of the Western blotting shown in *panel C*. *n* = 3, mean ± S.D. Statistical analysis was done using a one-way analysis of variance (ANOVA) with Bonferroni's multiple-comparison test. ***, *p* ≤ 0.001. *E*, Tau fibrils were treated for 4 h with either individual chaperones of the disaggregation machinery or all possible combinations, respectively. S and P fractions were separated by centrifugation, and Tau levels were analyzed by immunoblotting. Supernatant fractions of a representative experiment are shown. *F*, densitometric quantification of Tau in supernatant fractions compared with the total (S + P) of each sample of the experiment shown in *panel E*. *n* = 3, mean ± S.D. Statistical analysis was done using a one-way ANOVA with Bonferroni's multiple-comparison test. For clarity, only the significances to the –chaperone +ATP condition and between HSC70, DNAJB1 + ATP in the presence or absence of HSPA4 are indicated. **, *p* ≤ 0.01; ***, *p* ≤ 0.001.

We have previously shown that the human Hsp70 disaggregase comprised of HSC70, DNAJB1, and HSPA4 can disassemble α-synuclein fibrils *in vitro* (21). To investigate whether Tau fibrils can be disassembled by this chaperone system, we performed *in vitro* disaggregation assays (21) (Fig. 1*A*). Recombinant 1N3R Tau, harboring one N-terminal insertion and three microtubule binding repeats (Fig. S1), was assembled into fibrils, and fibril formation was verified by negative-stain transmission EM (TEM) (Fig. 1*B*). Tau fibrils were treated with the human Hsp70 disaggregation machinery (HSC70, DNAJB1, HSPA4) and subsequently centrifuged to separate larger fibrils from liberated smaller oligomers and monomers. The amount of Tau in supernatant (S) and pellet (P) fractions was analyzed by SDS-PAGE and immunoblotting (Fig. 1*C*). In the presence of the three chaperones and ATP, more than 40% of 1N3R Tau was detected in the supernatant fraction (Fig. 1*C* and *D*). In contrast, in the absence of ATP, the chaperone mix did not confer any significant disaggregation activity (Fig. 1*C* and *D*). The three components of the Hsp70 disaggregation machinery were also added individually and in all possible combinations to test their respective contribution to fibril disassembly (Fig. 1*E* and *F*). Treatment with single or pairwise combinations of chaperones did not promote a shift of Tau to the supernatant fraction, except HSC70 together with DNAJB1, which resulted in relocation of ∼28% of Tau to the supernatant (Fig. 1*E* and *F*). However, the combination of all three chaperones was required for the most efficient disaggregation reaction leading to ∼43% disassembly (Fig. 1*E* and *F*), indicating a critical role of the Hsp110-type NEF in Tau fibril disaggregation. In conclusion, the human HSC70/DNAJB1/HSPA4 disaggregation machinery efficiently disassembles a significant fraction of Tau fibrils *in vitro*.

### All six Tau isoforms can be disassembled by the disaggregation machinery

Human Tau has six different isoforms that are generated by alternative splicing (5) (Fig. S1). Whereas all isoforms were found in aggregates isolated from AD patients' brains, there are also isoform-specific tauopathies where amyloid deposits consist exclusively of either 3R or 4R Tau isoforms (4). For example, Tau filaments in Pick's disease contain only 3R Tau, whereas progressive supranuclear palsy is characterized by fibrils made entirely of 4R isoforms.

**Figure 2.**
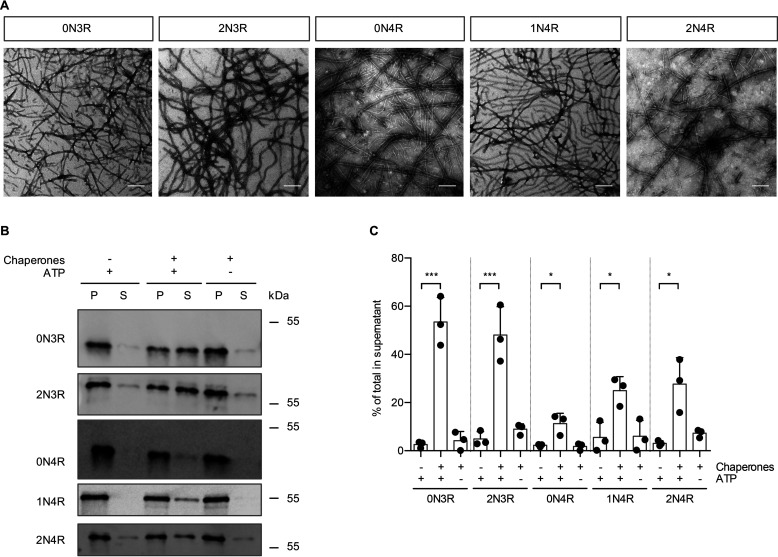
**In addition to 1N3R, the other five Tau isoforms are also substrates for the disaggregation machinery.** *A*, electron micrographs of negatively stained fibrils from recombinant Tau isoforms aggregated *in vitro*. *Scale bar*, 250 nm. *B*, recombinant fibrils formed from the five indicated Tau isoforms were incubated with the HSC70, DNAJB1, HSPA4 disaggregation machinery for 20 h at 30 °C. S and P fractions were separated by centrifugation and analyzed by Western blotting. *C*, Densitometric quantification of Tau levels in the experiment shown in *panel B*. *n* = 3, mean ± S.D. One-way ANOVA with Bonferroni's multiple comparison test was used. Significances are shown compared with the −Chaperone +ATP condition for each isoform. *, *p* ≤ 0.05; ***, *p* ≤ 0.001.

To test whether all six isoforms are substrates for the disaggregation machinery, recombinant fibrils of the other five Tau isoforms (0N3R, 2N3R, 0N4R, 1N4R, and 2N4R) (Fig. 2*A*) were subjected to disaggregation, and the reaction products were analyzed by differential centrifugation (Fig. 2*B*). Similar to 1N3R fibrils, fibrils formed by all other Tau isoforms could be disassembled by the human disaggregation machinery, although with varying efficiencies (Fig. 2*B* and *C*). In general, all 3R isoforms displayed higher disaggregation rates than their 4R counterparts, with 0N3R Tau fibrils being most efficiently disassembled (53%) and 0N4R fibrils being most resistant to chaperone-mediated disaggregation (11%). Overall, these results show that the Hsp70 disaggregase exhibits disaggregation activity toward all Tau variants and is not limited to fibrils assembled from a certain Tau isoform.

### Detergent-insoluble Tau extracted from cell culture and AD brain can be disaggregated

**Figure 3.**
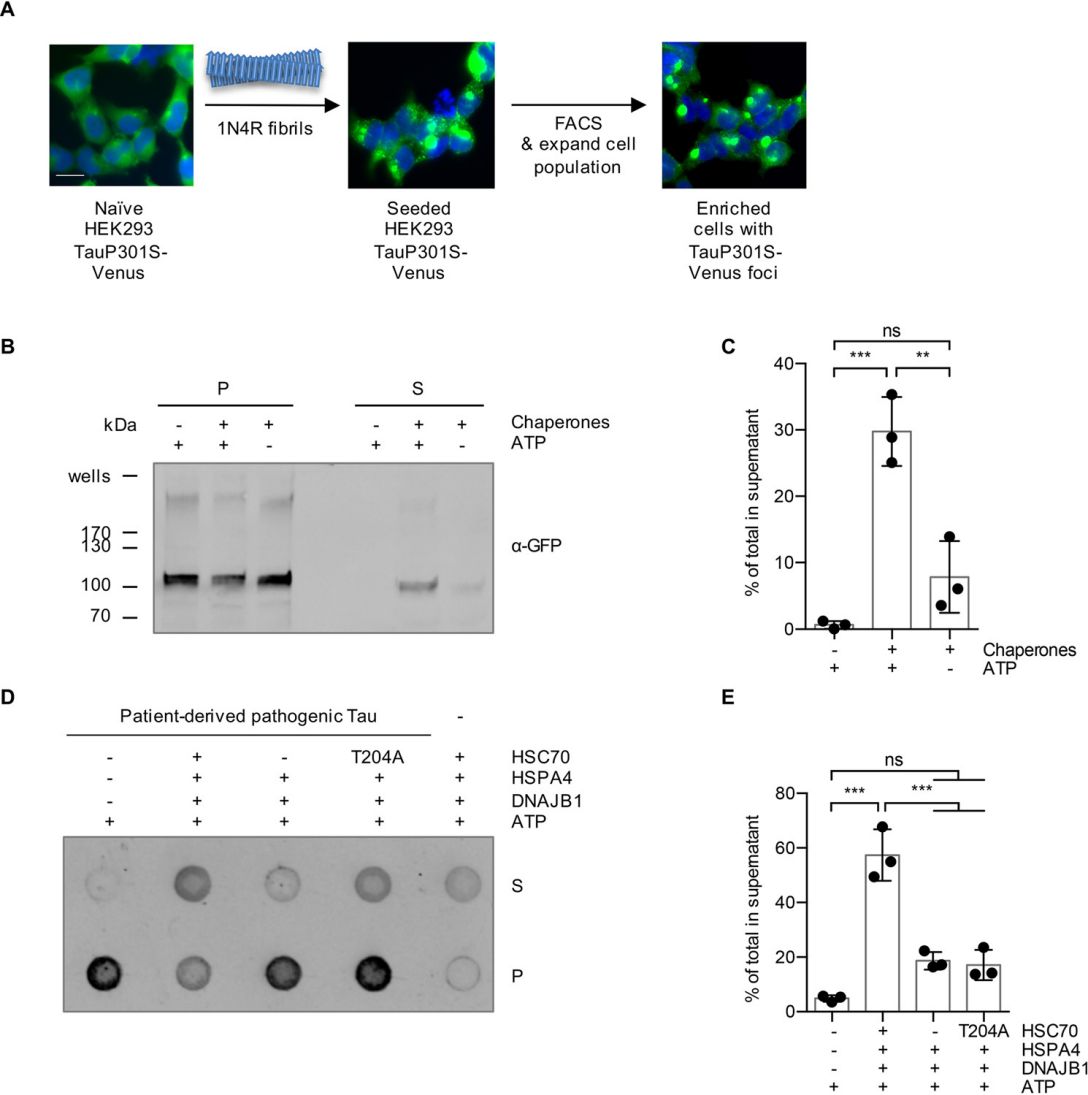
**Sarkosyl-insoluble TauP301S-Venus aggregated in a cell culture model and Tau aggregates extracted from AD brain are substrates for the disaggregation machinery.** *A*, a HEK293 0N4R TauP301S-Venus cell line was used to generate a cell population with Tau aggregates as outlined in the depicted workflow. Naïve cells were seeded with 1N4R fibrils, followed by the enrichment of focus-containing cells by FACS. A representative image of the seeded cell population after the FACS enrichment is shown. *Scale bar*, 20 μm. *B*, Sarkosyl-insoluble TauP301S-Venus material extracted from the seeded HEK293 cell population was subjected to *in vitro* disaggregation assays with the recombinant human Hsp70 disaggregation machinery (HSC70, DNAJB1, HSPA4) ± ATP for 20 h at 30 °C. S and P fractions were separated by centrifugation at 337,000 × *g* and analyzed by immunoblotting with an α-GFP antibody detecting TauP301S-Venus. *C*, densitometric quantification of TauP301S-Venus in S fractions compared with the total (S + P) of the Western blot shown in *panel B*. *n* = 3, mean ± S.D. One-way ANOVA with Bonferroni's multiple comparison test. *ns*, not significant; ***, *p* ≤ 0.001. *D*, the Sarkosyl-insoluble fraction extracted from an AD brain was subjected to *in vitro* disaggregation assays for 20 h at 30 °C. The samples were treated either with the complete disaggregation machinery (HSC70, DNAJB1, HSPA4), with only DNAJB1 and HSPA4, omitting HSC70, or with DNAJB1 and HSPA4 together with an ATPase-defective mutant HSC70 (T204A). S and P fractions were separated by centrifugation at 150,000 × *g* and analyzed by dot blotting with the α-Tau antibody HT7. *E*, densitometric quantification of the dot blot shown in *panel D*. The percentage of Tau in S fractions compared with the total (S + P) was calculated for each sample. The background signal of chaperones without patient material was subtracted. *n* = 3, mean ± S.D. One-way ANOVA with Bonferroni's multiple comparison test was used. *ns*, not significant; ***, *p* ≤ 0.001.

Amyloid aggregates formed *in vivo* might possess different properties than *in vitro* aggregated fibrils, as posttranslational modifications or coaggregation with other endogenous proteins could affect the overall structural arrangement (22). To investigate whether Tau aggregates formed in cells are clients of the human Hsp70 disaggregation machinery, we made use of a HEK293 cell model of Tau aggregation (23). This cell line constitutively overexpresses Venus-tagged full-length P301S mutant 0N4R Tau (0N4R TauP301S-Venus), which remains Sarkosyl soluble under normal growth conditions and was successfully used as a biosensor for Tau seeding (23). After treating the cells with recombinant fibrils from the 1N4R Tau isoform, we specifically enriched the seeded cells through flow cytometry sorting and expanded them to provide a source of Tau aggregates that were formed in cells (Fig. 3*A*). The Sarkosyl-resistant material was extracted from the seeded cells and subjected to *in vitro* disaggregation assays. In the presence of HSC70, DNAJB1, HSPA4, and ATP, 30% of the TauP301S-Venus was recovered in the supernatant fraction following centrifugation (Fig. 3*B* and *C*). Next, we evaluated whether patient-derived Tau can also be disassembled by the Hsp70 disaggregation machinery. We prepared Tau filaments from Braak stage VI AD cortex gray matter using Sarkosyl extraction and extensive ultracentrifugation steps as previously described (24) (Fig. S2). Treating Tau filaments prepared in this manner with the disaggregation machinery *in vitro* released 57% of Tau into the supernatant fraction (Fig. 3*D* and *E*). Together, these results demonstrate that the human Hsp70 disaggregation machinery is capable of disassembling Sarkosyl-insoluble Tau, which was aggregated in a human cell culture model, as well as pathological Tau species extracted from the brain of an AD patient.

### Class B J-domain proteins mediate disaggregation

Hsp70 substrate specificity is mediated by J-domain proteins that recognize chaperone clients and deliver them to Hsp70 (13). Humans encode more than 40 different J-domain proteins, subdivided into structural classes A, B, and C, with distinct substrate specificities and cellular localization (13, 15). Several class A and B J-domain proteins are differentially regulated in the brain both during aging and in neurodegenerative diseases (25). In particular, the class A cochaperone DNAJA1 as well as the class B cochaperone DNAJB4 are upregulated in patients with AD, PD, and Huntington's disease (HD) compared with age-matched controls (25). These findings point to a potential role of these cochaperones in regulating proteostasis in the context of protein-misfolding diseases.

**Figure 4.**
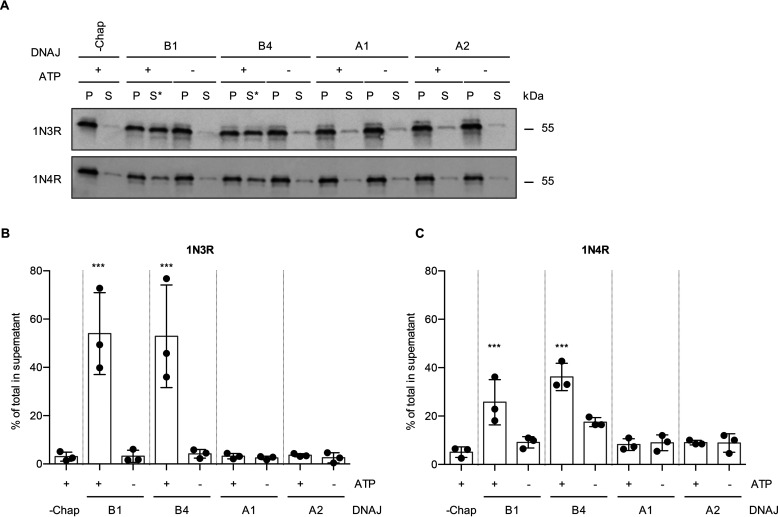
**Class B J-domain proteins mediate Tau disaggregation.** *A*, Tau fibrils made up from 1N3R or 1N4R were treated with HSC70, HSPA4, and different HSP40 class A and class B J-domain proteins, respectively. Subsequently, S and P fractions were separated by centrifugation and Tau levels were analyzed by Western blotting. Asterisks indicate successful disaggregation reactions. *B*, densitometric quantification of 1N3R Tau levels in S fractions compared with the total (S + P) as shown in *panel A*. *n* = 3, mean ± S.D. One-way ANOVA with Bonferroni's multiple-comparison test was used. Only significances to the −Chaperone +ATP condition are indicated. ***, *p* ≤ 0.001. *C*, densitometric quantification of 1N4R Tau levels in S fractions compared with the total (S + P) as shown in *panel A*. *n* = 3, mean ± S.D. One-way ANOVA with Bonferroni's multiple comparison test was used. Only significances to the –Chaperone (−*Chap*) +ATP condition are indicated. ***, *p* ≤ 0.001.

Therefore, we investigated whether these J-domain proteins could also serve in combination with HSC70 and HSPA4 to disaggregate Tau fibrils (Fig. 4*A*). The class B J-domain protein DNAJB4 was equally capable of promoting disaggregation of recombinant 1N3R Tau fibrils as DNAJB1, both shifting ∼50% of Tau to the supernatant fraction (Fig. 4*A* and *B*). Although 1N4R fibrils again were less susceptible to disassembly by the Hsp70 disaggregation machinery than 1N3R fibrils, DNAJB4 could also substitute for DNAJB1 and mediated ∼36% disaggregation compared with ∼26% for DNAJB1 (Fig. 4*A* and *C*). The two class A J-domain proteins tested here, DNAJA1 and DNAJA2, did not enable disaggregation of either 1N3R or 1N4R Tau fibrils (Fig. 4*A*–*C*). Moreover, DNAJA2 did not promote the disaggregation of any other Tau isoform (Fig. S3*A*). In conclusion, the class B J-domain protein DNAJB4, which is closely related to DNAJB1 (Fig. S3*B* and *C*), also enabled efficient disaggregation of amyloid Tau fibrils, whereas the class A J-domain proteins could not assist their disassembly.

### Tau disaggregation yields monomeric and small oligomeric seeding-competent species

The products of the disaggregation reaction could consist of multiple protein species, such as monomers, small oligomers, and other fibril fragments with intermediate lengths. Furthermore, it is not yet clear whether chaperone-mediated disaggregation of amyloid fibrils is advantageous or disadvantageous. A complete resolubilization and refolding of Tau into monomers is considered beneficial, whereas the production and accumulation of fibrillar intermediates is considered disadvantageous, as the latter could contribute to the propagation of Tau aggregates through the continuous production of new seeds. Therefore, it is important to analyze the products of the disaggregation reaction more closely.

**Figure 5.**
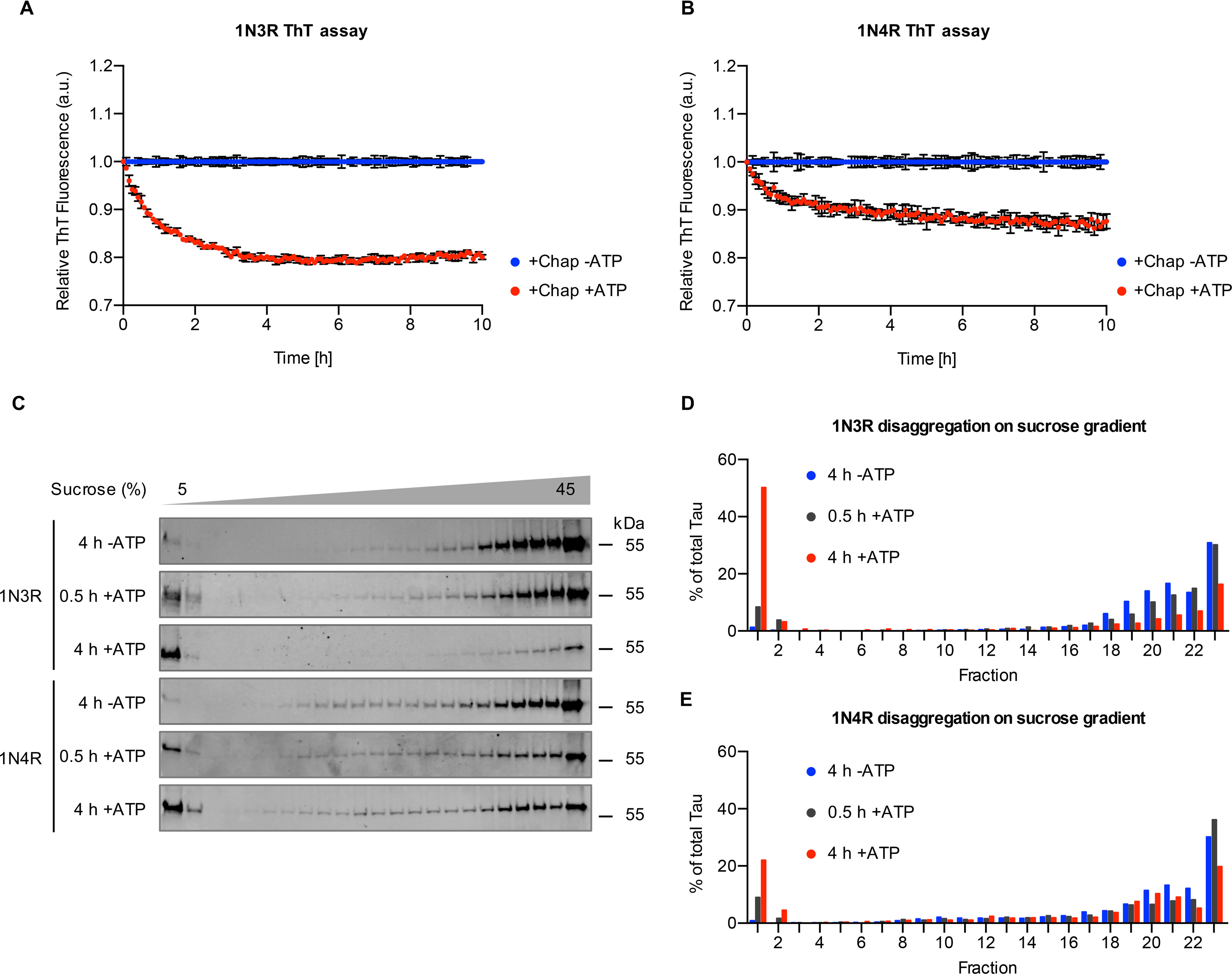
**The disaggregation reaction generates low-molecular-weight species.** *A* and *B*, 1N3R (*A*) and 1N4R (*B*) Tau fibrils were treated with chaperones in the presence or absence of ATP. Thioflavin T (ThT) fluorescence was monitored over time as a readout of amyloid content. Per condition, three independent disaggregation reactions were measured (*n* = 3, mean ± S.D.). *C*, Tau fibrils were treated with the disaggregation machinery for 30 min or 4 h at 30 °C and subsequently centrifuged over a 5–45% sucrose gradient. Representative blots of three (1N3R) and two (1N4R) replicates are shown. Fractions were collected manually, and Tau levels were analyzed by immunoblotting. *D* and *E*, densitometric quantification of 1N3R disaggregation (*D*) and 1N4R disaggregation (*E*) of the experiment shown in *panel C*. The amount in each fraction was calculated as a percentage of the total amount of Tau in all fractions.

To monitor the disaggregation dynamics, we determined the quantity of amyloid structures during chaperone-mediated disaggregation by measuring the fluorescence of the amyloid-specific dye thioflavin T (ThT) over time. Incubation of both 1N3R (Fig. 5*A*) and 1N4R (Fig. 5*B*) Tau fibrils with the disaggregation machinery and ATP resulted in a decrease in ThT fluorescence of 20% and 10% within 4 h, respectively. After that, the ThT fluorescence plateaued over the course of the measurement. In the absence of ATP, the ThT fluorescence remained stable over time. This observation reflected the results obtained by differential centrifugation for the two Tau isoforms (Fig. 1*C* and *D* and 2*B* and *C*). However, the disaggregation efficiency determined by reduction in ThT fluorescence was, in general, lower than the efficiencies obtained by the sedimentation assay and subsequent immunoblotting.

To characterize the composition of the disaggregated Tau material in more detail, we performed rate-zonal centrifugation, in which Tau assemblies are separated according to their size. We first performed control experiments with untreated monomeric and fibrillar Tau, respectively (Fig. S4). After 3 h of centrifugation over a 5–45% sucrose gradient, monomeric recombinant 1N3R and 1N4R Tau remained in the first two low-density fractions, whereas untreated fibrils migrated to the higher density fractions (Fig. S4). Next, 1N3R and 1N4R Tau fibrils were analyzed after 30 min and 4 h of chaperone treatment. These time points were chosen based on the disaggregation kinetics followed using ThT binding, reflecting the intermediate and endpoints of the disaggregation reaction, respectively (Fig. 5*A* and *B*).

In the presence of HSC70, DNAJB1, HSPA4, and ATP, an increasing amount of Tau was detected in the low-density fractions over the course of the disaggregation reaction, whereas the amount of Tau in all middle- and high-density fractions was reduced (Fig. 5*C*–*E*). Fibrils incubated for 4 h with chaperones but without ATP behaved like untreated fibrils and moved to higher density fractions during the centrifugation, and no shift of Tau to lower density fractions was observed (Fig. 5*C*–*E* and Fig. S4). We did not detect any buildup of Tau in intermediate fractions during the disaggregation reaction (Fig. 5*D* and *E*). This finding was also in line with our EM analysis, where we did not observe widespread fibril fragmentation (Fig. S5). Instead, most of the Tau protein liberated by the disaggregation machinery shifted to the low-density fractions in the sucrose gradients, suggesting that predominantly monomeric or small oligomeric Tau species were released.

**Figure 6.**
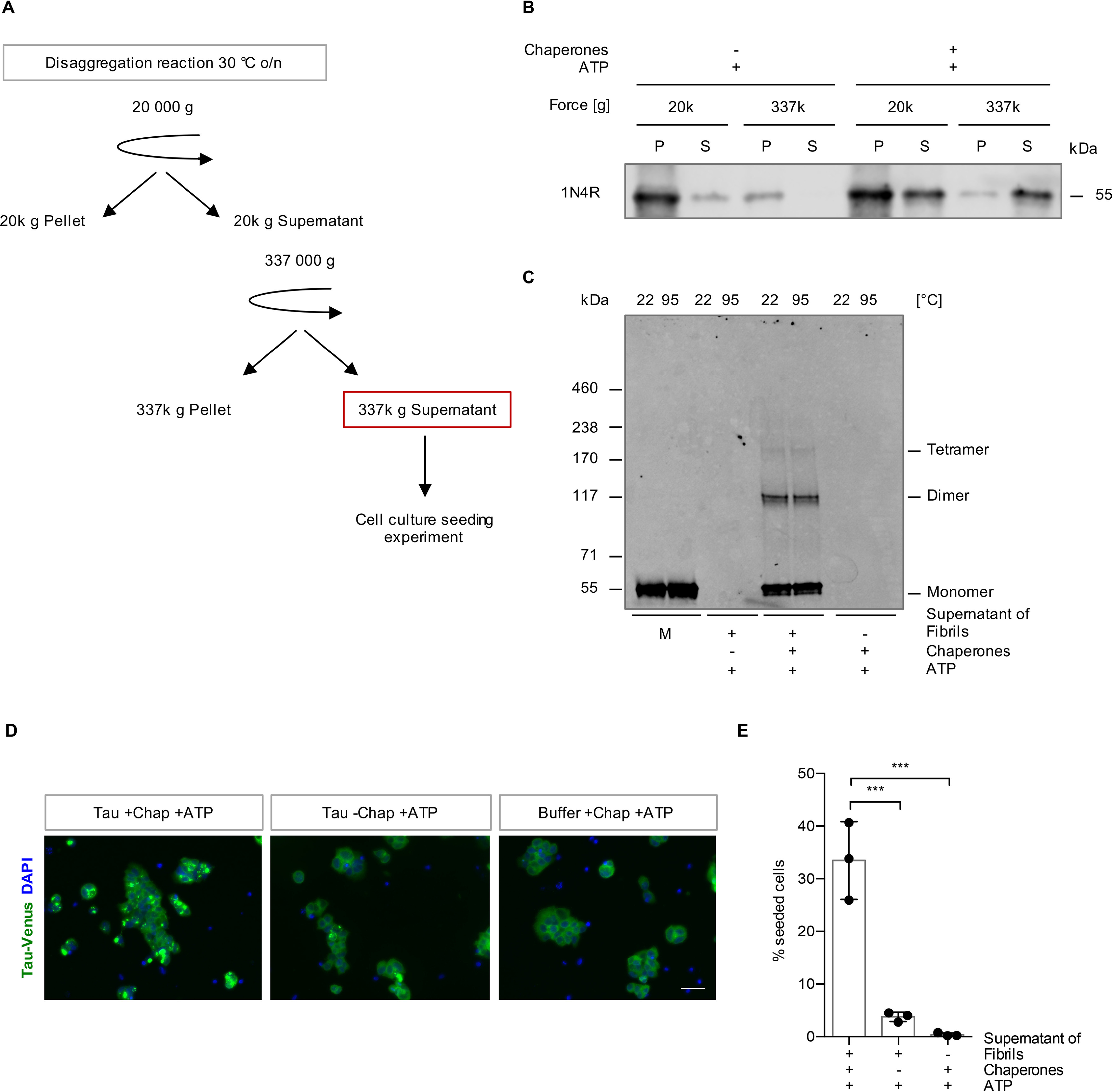
**The disaggregation reaction liberates seeding-competent Tau species.** *A*, experimental setup of the cell culture seeding assay. Differential centrifugation of 20,000 × *g* followed by ultracentrifugation at 337,000 × *g* was applied to isolate the Tau material that was liberated by the action of the Hsp70 disaggregation machinery. The 337,000 × *g* S fractions were tested for their seeding capacity in a HEK293 cell culture model for Tau aggregation. *B*, Tau levels in the S and P fractions that were collected during the differential centrifugation steps shown in *panel A* were analyzed by immunoblotting. *C*, the 337,000 × *g* S fractions of disaggregated Tau and monomeric Tau were incubated with 2% SDS at room temperature or 95 °C and run on Tris acetate–SDS-PAGE. The samples were analyzed by immunoblotting. *D*, maximum intensity projections of fluorescence microscopy z-stacks of TauP301S-Venus HEK293 cells seeded with the 337,000 × *g* supernatant fraction after the disaggregation reaction. Cells were fixed 24 h after the treatment. *Scale bar*, 50 μm. *E*, quantification of cells containing TauP301S-Venus foci. *n* = 3 replicates with 288–460 cells per condition in each replicate, with mean ± S.D. Statistical analysis was performed using a one-way ANOVA with Bonferroni's multiple-comparison test. ***, *p* ≤ 0.001.

To further characterize these low-molecular-weight Tau species, we next subjected Tau fibrils with and without chaperone treatment to sequential centrifugation (Fig. 6*A*). In untreated 1N4R Tau fibril preparations, about 6% of Tau was found in the 20,000 × *g* supernatant (Fig. 2*B*). However, these Tau species sedimented at 337,000 × *g*, whereas the Tau material that was released by disaggregation remained in the supernatant, hinting at a smaller particle size of these species (Fig. 6*B*). Hence, the 337,000 × *g* supernatant contains Tau species specifically produced by the action of the Hsp70 disaggregation machinery, which are not present without chaperone treatment. Tris acetate–SDS-PAGE revealed that the disaggregated material contained monomeric as well as oligomeric Tau species with an apparent molecular weight compatible with that of dimeric and tetrameric Tau (Fig. 6*C*). The latter migrated as distinct bands with apparent molecular weights of 100 kDa and 200 kDa in the gel and could not be dissolved by incubating in 2% SDS at 22 °C or 95 °C (Fig. 6*C*).

Finally, we evaluated the seeding propensity of this fraction of Tau released by the Hsp70 disaggregase in the TauP301S-Venus HEK293 cell line (23). The TauP301S-Venus-expressing HEK293 cells were exposed to this material for 24 h, and the percentage of focus-containing cells was evaluated by fluorescence microscopy. The 337,000 × *g* supernatant of Tau fibrils incubated with the disaggregation machinery and ATP triggered focus formation in ∼33% of cells. None of the supernatants of untreated fibrils, the chaperones of the disaggregation machinery and ATP, buffer with ATP (Fig. 6*D* and *E*), and naïve monomeric Tau (Fig. S6*A*) induced focus formation. Moreover, treatment with 1N3R fibrils also did not lead to the accumulation of visible TauP301S-Venus deposits, indicating that focus formation depends on sequence-specific templated misfolding (Fig. S6*B*). We further assessed the longitudinal propagation of the seeded Tau species, *i.e.* the inheritance of Tau foci in cells transiently exposed to the Tau released by chaperone action. These Tau foci persisted in daughter cells during subsequent cell passages (Fig. S6*C*), demonstrating that Tau species generated by the Hsp70 disaggregation machinery were not only seeding competent but also able to induce aggregate species that stably persisted in dividing cells.

In conclusion, Tau fibril disassembly by the human Hsp70 disaggregation machinery did liberate monomeric, as well as dimeric and tetrameric Tau species, which were seeding competent and induced self-propagating Tau conformers in an HEK293 cell culture model for Tau aggregation.

## Discussion

It is well established that the cellular network of molecular chaperones assists in all aspects of protein quality control, from folding of newly synthesized peptides to the disassembly of protein aggregates and degradation of terminally misfolded proteins (1, 10, 11). Chaperones thereby affect many disease states, which is why chaperone-based therapies could be a promising treatment approach. However, it remains poorly understood to what extent chaperones are capable of disassembling already existing amyloids, given their high thermodynamic stability (26). Only for α-synuclein and HTTExon1Q_48_ has it been established that the human Hsp70 machinery can disassemble preformed fibrils *in vitro* (21, 27). Here, we investigated the broader role of this machinery in amyloid biology by testing its potential to process aggregates of amyloidogenic Tau isolated from AD brain, from a cell culture model, or produced *in vitro* and by characterizing more precisely the products of chaperone-mediated Tau disaggregation. This is particularly important because Tau aggregation is central to the most prevalent human neurodegenerative diseases, including AD, and also plays a role in traumatic brain injuries (4, 28).

We show that the Hsp70 disaggregation machinery is capable of disassembling *in vitro* aggregated Tau amyloid fibrils as well as Sarkosyl-resistant Tau aggregates formed in cell culture. Importantly, even pathological Tau extracted from human AD brain tissue was disaggregated, demonstrating that this chaperone machine can dissociate disease-relevant protein species. Tau disaggregation resulted in a rapid accumulation of low-molecular-weight Tau species (Fig. 5*C* and 6*C*). Further characterization of the liberated Tau pool revealed that it contained mostly monomeric and also some oligomeric species with apparent molecular weights of ∼100 kDa and ∼200 kDa, compatible with that of dimeric and tetrameric Tau, respectively. Intriguingly, this material was still seeding competent, as it induced longitudinal self-propagating aggregates of a stably expressed full-length Tau reporter in a HEK293 cell culture model, implying that chaperone-mediated Tau disaggregation is not beneficial per se but may be involved in the prion-like propagation of Tau pathology. Therefore, it will be important to further investigate the oligomerization state of the liberated Tau material under native conditions and to unravel the exact nature of the seeding-competent pool.

Amyloid fibrils share a common core structure consisting of a characteristic β-sheet-rich conformation (29). Although exhibiting a very similar architecture, fibrils composed of α-synuclein, HTTExon1Q_48_, and Tau will display different surface properties, as they do not share any sequence homology (30–32). Nevertheless, the Hsp70 disaggregation machinery can disassemble amyloid fibrils composed of each of these proteins, albeit with varying efficiencies. While we could detect ∼60% disassembly of Tau fibrils in this study and up to 80% disaggregation efficiency for α-synuclein fibrils in earlier work (21), Scior *et al*. (27) report that ∼30% of HTTExon1Q_48_ could be solubilized by the human Hsp70 disaggregase. Overall, these studies highlight the versatility of the disaggregation machinery to process various amyloid substrates.

Still, despite overall structural similarity, conformational variations of the amyloid structure formed by a given protein, including Tau, are known to exist and to affect the pathology of the associated disease (30–32). This variability could make some fibrils more resistant to chaperone action. Indeed, we observed differences in disaggregation efficiencies between the six distinct Tau isoforms. Fibrils comprised of 0N4R Tau were most resistant to disaggregation, resulting in only 10% disaggregated material compared with up to 64% obtained with the other isoforms. 0N4R Tau fibrils also remained intact after the addition of the Hsp70 disaggregation complex in another study (17). Fibrils assembled from 3R Tau isoforms were disassembled to a greater extent than those made of the 4R isoforms (Fig. 1*D* and 2*C*). *In vitro*-assembled fibrils from 2N3R and 2N4R Tau vary in their architectures (33). The ordered core of 2N3R Tau fibrils comprises the R3 repeat of two parallel Tau molecules, whereas 2N4R Tau fibrils adopt several conformations with a core consisting of R2 and R3 β-strands of the same molecule (33). Intriguingly, these differences in fibril architecture may lead to a different stability of 3R and 4R Tau fibrils, which may explain their varying susceptibility to the human Hsp70 disaggregation machinery. Alternatively, or additionally, the kinetics of chaperone binding may vary between isoforms, leading to different disaggregation efficiencies. This hypothesis is supported by the fact that Hsp70 shows an isoform-dependent difference in both binding affinity and aggregation prevention of monomeric 3R and 4R Tau variants (19, 34). However, the assembly of higher order structures could create new chaperone binding sites, which might alter binding constants, and cochaperones are known to affect the affinity of Hsp70 to its substrates. Thus, whether differences in chaperone binding play a role here remains an open question and requires further investigation.

Intriguingly, the Hsp70 disaggregase also solubilized Sarkosyl-insoluble Tau material extracted from an AD patient's brain very efficiently (Fig. 3*D* and *E*). Tau aggregates isolated from AD brains incorporate all six Tau isoforms and adopt an ultrastructural arrangement distinct from that of *in vitro*-assembled recombinant Tau (33, 35). In addition, posttranslational modifications or coaggregating interactors can alter the amyloid structure or affect chaperone interactions (36, 37). Hence, these data demonstrate the capacity of the Hsp70 disaggregase to process a broad range of physiologically relevant substrates and underlines the versatility of this chaperone system. Because of the structural differences between the Tau amyloid conformers observed in different tauopathies (33), it would be of interest to investigate the chaperone disaggregation capacity on Sarkosyl-insoluble material from other tauopathies besides AD, especially to compare 3R- and 4R-only diseases.

We never observed a disaggregation efficiency greater than 64%. This could be because of the mixture of different Tau conformers in our fibril preparation that is subjected to disaggregation. A subset thereof might be readily disaggregated, whereas other fibril types might be completely resistant. However, this is unlikely, as we could not detect the disappearance of a certain type of fibril after chaperone treatment by TEM. Alternatively, the amyloid equilibrium might prevent disaggregation completion. Amyloid fibrils exist in equilibrium with monomeric species in a solution with the amyloid state highly favored. Disaggregation produces both monomeric and oligomeric species that can reaggregate over the course of the disaggregation reaction. The exact percentage of disaggregation likely depends on the kinetics of disaggregation in relation to the kinetics of (re-)aggregation and seeding by disaggregation products, which in turn depends on the respective fibril type. This ultimately leads to a conformation- and assay-specific disaggregation efficiency until equilibrium is reached. This might also explain why we repeatedly observed a lower disaggregation efficiency in the ThT assay compared with the supernatant pellet assay. Regular shaking of the sample during the ThT assay (in contrast to the normal disaggregation reaction, where the samples remain unshaken) could facilitate seeding and reaggregation and shift the amyloid equilibrium toward fibrillar forms. In addition, the binding of ThT to amyloid structures is not trivial, and studies have shown that ThT has varying affinities to distinct conformers and assemblies (38, 39). Therefore, the signal is not strictly proportional to the amount of fibrils and may introduce a bias. It is conceivable that fibril assemblies, which exhibit a lower ThT signal, might be preferentially disaggregated.

J-domain protein cochaperones are known to confer substrate specificity to the Hsp70 machinery (13). Our data revealed that the class B J-domain proteins, DNAJB1 and DNAJB4, enabled HSC70 to disaggregate Tau fibrils. In contrast, neither of the two major cytosolic class A J-domain proteins we tested mediated Tau disaggregation. This is consistent with our past observations with α-synuclein fibrils (21). Class A J-domain proteins appear to be successful in the prevention of aggregation of monomeric Tau (17), whereas class B J-domain proteins are capable of recognizing preformed Tau fibrils as substrates and recruiting the Hsp70 disaggregation machinery to this kind of polymeric client. However, it remains to be addressed why DNAJB1 and DNAJB4 are so potent at supporting the disassembly of amyloid fibrils.

An intriguing difference between the disassembly reactions of α-synuclein and Tau is that Tau disaggregation did not lead to a significant increase of intermediate-length fragments but primarily produced low-molecular-weight species instead. Despite containing predominantly monomeric and a few oligomeric Tau species, this material had significant seeding propensity in a biosensor HEK293 cell model expressing full-length TauP301S and induced the formation of self-propagating Tau species (Fig. 6*D*). This implies that low-molecular-weight species or even monomeric Tau are seeding competent, which is in line with a recent study showing that fibril-derived Tau monomers exhibit seeding activity (40). Hence, monomeric or small oligomeric Tau liberated from fibrils by chaperone action might still maintain a seeding-competent conformation that is different from that of naïve monomeric Tau.

Because amyloid structures propagate by seed-induced templated misfolding (41), chaperone-mediated disaggregation might be detrimental *in vivo* by generating additional seeds that can sequester more Tau into amyloid aggregates, thereby accelerating disease progression. In support of this idea, we recently showed that the Hsp70 disaggregation machinery contributes to prion-like spreading of amyloidogenic proteins in *Caenorhabditis elegans* (*C. elegans*) (42). Compromising the disaggregation machinery by knocking down the *C. elegans* homolog of HSPA4 reduced disaggregation of α-synuclein and polyglutamine (Q_35_) aggregates, thereby decreasing their amplification and toxicity. Hence, it is tempting to speculate that the chaperone-mediated disaggregation of Tau plays a similar role in the prion-like propagation of Tau pathology throughout the brain.

Interestingly, two alternative systems have been recently described that are also able to dissolve Tau fibrils *in vitro* independently of Hsp70. The human peptidyl-prolyl isomerase cyclophilin 40 reaches efficiencies similar to those of the Hsp70 disaggregase, reducing ThT-positive Tau species by 50% (43). In addition, proteasomes isolated from HEK cells were shown to disintegrate Tau fibrils *in vitro*, thereby releasing small fragments and oligomeric species (44). Thus, it seems that there might be several parallel cellular systems that can disintegrate and fragment amyloid fibrils. However, their respective significance for the prion-like propagation and toxicity of Tau aggregates *in vivo* has yet to be clarified.

Chaperone activity is commonly believed to decline during aging and in the context of neurodegenerative diseases. However, this view is too simplified. Rather, a recent study revealed that an imbalance occurs where individual members are deregulated, with some being up- and others downregulated in the aging or diseased human brain (25). Brehme *et al*. (25) analyzed several previously published gene expression datasets from brains of aged people (45, 46) and patients suffering from AD (superior frontal gyrus [47]), HD (prefrontal cortex [48]), or PD (substantia nigra [49]). This meta-analysis revealed that DNAJB4 expression is repressed during physiological aging, whereas it is slightly upregulated in patient brains. Because DNAJB4 promoted disaggregation as efficiently as DNAJB1 in our study (Fig. 4), this cochaperone could directly link enhanced disaggregation activity to amyloid propagation in neurodegeneration. Further research on the regulation of DNAJB4 levels in both glial and neuronal cells during aging and neurodegeneration will help to identify a potential contribution to disease etiology.

Another important aspect is that Tau disaggregation (by chaperones or other molecular machines) does not occur in isolation and might be coupled to protein degradation via the proteasome or autophagy in the cellular context. These pathways might be more potent in degrading smaller oligomers and monomers instead of larger fibrils, which would render disaggregation activity beneficial overall. Hence, further studies are necessary to clarify the exact role of the Hsp70 disaggregation machinery in Tau amyloid aggregation and toxicity *in vivo*. Such studies will allow evaluating the therapeutic potential of the Hsp70 chaperone machinery in tauopathies.

## Experimental procedures

All chemicals were purchased from Sigma-Aldrich or Carl Roth unless stated otherwise.

### Purification of recombinant proteins

The six isoforms of full-length human Tau, *e.g.* 0N3R, 0N4R, 1N3R, 1N4R, 2N3R, and 2N4R Tau, were expressed and purified as described previously (50). Tau protein concentration was determined spectrophotometrically using an extinction coefficient at 280 nm of 7450 m^−1^cm^−1^. Aliquots of pure Tau isoforms (100 μm) were stored at −80 °C.

Monomeric Tau was used as a control in several experiments of this study. Aliquots were stored at −80 °C. To remove aggregates that might have formed during the freeze-thaw process, samples were centrifuged at least at 100,000 × *g* immediately before using the supernatant for any experiment.

The human chaperone HSC70, the ATPase-defective mutant HSC70 T204A (51, 52), DNAJB1, DNAJA1, DNAJA2, and HSPA4 were purified as previously published (21, 53, 54). Briefly, N-terminally His6-Sumo-tagged proteins were expressed in *E. coli* BL21(DE3) and affinity purified using Protino Ni-NTA agarose (Macherey-Nagel). Subsequently, the tag was cleaved off by Ulp-1 digest and both the His6-Sumo tag and the His-tagged Ulp1 were removed by a second Ni^2+^ affinity purification step. The proteins were further purified by size exclusion chromatography on a Superdex200 16/60 column (GE Healthcare).

The human DNAJB4 DNA sequence (The ORFeome Collaboration [55], DKFZ) was cloned into a pCool6 vector with an N-terminal His6-Sumo tag generated previously (56) and expressed at 16 °C. Further purification steps were performed by following the protocol stated above. Aliquoted proteins were stored at −80 °C.

### In vitro aggregation of recombinant Tau

Fibrillation of the six Tau isoforms was achieved at 40 μm in the presence of 10 μm heparin by shaking 0.5-ml aliquots of solution at 37 °C in an Eppendorf Thermomixer set at 600 rpm for 4 days. At steady state, an aliquot from each assembling reaction was spun for 35 min at 20 °C and 50,000 rpm (113,000 × *g*), and the amount of Tau in the supernatant was assessed spectrophotometrically and by SDS-PAGE to further demonstrate assembly completion. The amount of fibrillar Tau was estimated by subtraction of the soluble fraction remaining after centrifugation from the initial concentration.

### Immunohistochemistry of patient brain sample

Deparaffinized 10-μm sections of the primary visual cortex were obtained from a case of Braak stage IV AD (Cambridge Brain Bank). Sections were subject to antigen retrieval in 98% formic acid for 5 min followed by 4% aqueous hydrogen peroxide to block endogenous peroxidases. Sections were then rinsed with tap water and PBS before being blocked with normal rabbit serum (Dako) in 20% PBS. Sections were then incubated with antibody to phosphorylated Tau protein (1:500, AT8, MN1020, Thermo) for 1 h. After rinsing for 5 min in PBS, they were incubated with secondary antibody (1:200, rabbit anti-mouse, Dako) for 30 min. After rinsing for 5 min in PBS, they were incubated in avidin-biotin complex (ABC, Vector Laboratories) for 30 min before being developed with diaminobenzidine (DAB, Vector Laboratories). Slides were then lightly counterstained with hematoxylin. Digital images were obtained using a camera (Infinity 2, Lumenera) attached to a microscope (Olympus BX53).

### Preparation of Sarkosyl-insoluble Tau from AD brain

Tau filaments were obtained from anonymized postmortem tissue of a 79-year-old male severe AD patient from the Cambridge Brain Bank under National Research Ethics Service approval number 10/H0308/56. The studies abide by the principles of the Declaration of Helsinki and were donated via the Cambridge Brain Bank. Pathological criteria were Braak stage VI, CERAD III, Thal II, Lewy body Braak stage 0, and TDP negative. 2 g of cortical gray matter was extracted according to a modified version of the method of Guo *et al*. (24). Briefly, fresh-frozen cortical gray matter was homogenized in 9 volumes of extraction buffer (10 mm Tris-HCl [pH 7.5], 0.8 m NaCl, 10% sucrose, 1 mm EDTA, 0.1 mm PMSF, 0.1% Sarkosyl, 2 mm imidazole, 1 mm NaV, 1 mm NaF, 2 mm DTT, Complete Ultra EDTA-free protease inhibitor mixture [Roche]) using a VelociRuptor V2 homogenizer and tubes prefilled with 2.8-mm acid-washed stainless steel beads. Homogenate was spun at 10,000 × *g* for 10 min at 4 °C and filtered through a 50-μm cell strainer. The pellet was reextracted with a further 4.5 volumes of extraction buffer and homogenized and clarified as above. Filtered supernatants were combined, and Sarkosyl was added to a final concentration of 1% before stirring at 100 rpm for 1 h. Samples were then subjected to ultracentrifugation at 100,000 × *g* for 75 min at 4 °C. The supernatant was separated from the pellet, and the latter was rinsed with PBS before resuspension and vortexing to break it apart. The resuspended pellet was further diluted in PBS and then centrifuged at 130,000 × *g* for 1 h at 4 °C. The resulting pellet was resuspended in 100 µl per gram gray matter and broken apart by 16 h of agitation at room temperature and passing through 18-, 23-, and 26-gauge needles. The resuspended pellet was sonicated (Hielsher S26D11X10 Vial-Tweeter Sonotrode at settings A 100%, C 50%, and 200 Ws). The sample was then centrifuged at 100,000 × *g* for 40 min at 4 °C. The pellet was resuspended again in 50 µl PBS per gram of gray matter and subjected to breaking apart using needles and sonication as above. Finally, the sample was subjected to a clearing spin at 10,000 × *g* at 4 °C. The concentrated Tau filaments were stored at −80 °C prior to use.

### In vitro disaggregation assay

*In vitro* disaggregation reactions were performed as previously described (21), with minor adjustments. Briefly, recombinant Tau fibrils aggregated *in vitro* (2 μm) or Sarkosyl-insoluble material extracted from seeded TauP301S-Venus HEK293 cells or extracted from AD patient brain material were incubated with the disaggregation machinery (HSC70 [4 μm], DNAJB1 [2 μm], and HSPA4 [0.2 μm]) in disaggregation buffer (50 mm Hepes-KOH [pH 7.5], 50 mm KCl, 5 mm MgCl_2_, and 2 mm DTT) at 30 °C for the indicated times. For +ATP conditions, 2 mm ATP and an ATP regeneration system (4.5 mm phospho-enolpyruvate, 20 ng/ml pyruvate kinase [Sigma-Aldrich]) were added to the reaction. For −ATP conditions, both ATP and the ATP regeneration system were omitted. After the indicated incubation times, samples were centrifuged for 30 min at 20,000 × *g* or 337,000 × *g* at 4 °C or for 75 min at 150,000 × *g* at 4 °C. Tau levels in supernatant (S) or pellet (P) fractions were subsequently analyzed by SDS-PAGE or dot blot and immunoblotting.

### ThT disaggregation assay

For kinetic analysis of the disaggregation reaction, 50-μl samples were prepared as described above but including 20 μm ThT. Buffer without Tau fibrils or chaperones was used as a blank control. The measurement was performed in sealed (Microseal B adhesive sealer, Bio-Rad) black 96-well clear-bottom plates (flat bottom, nonbinding surface, Corning) at 30 °C using a FLUOstar Omega plate reader (BMG Labtech). ThT fluorescence was recorded by bottom reading every 5 min with excitation/emission wavelengths set to 440/480 nm. Before each acquisition cycle, the plate was shaken for 10 s at 300 rpm. The data recorded from each well were blank corrected by subtracting the mean of the corresponding background control (buffer only or buffer plus chaperones) at each time point. Next, the relative fluorescence of each well and time point to the mean of the non-disaggregating conditions, chaperone-ATP, was calculated. Finally, to obtain the relative ThT fluorescence over time for each well, the data were normalized to the *t* = 0 min fluorescence value of each well.

### Rate-zonal centrifugation

Continuous 5–45% sucrose gradients (in 50 mm Hepes-KOH [pH 7.5], 50 mm KCl, 5 mm MgCl_2_) were formed in 12-ml open-top polyclear centrifuge tubes (Seton Scientific) on the *ip* Gradient Station (BioComp Instruments). Tau fibrils were incubated with the disaggregation machinery at 30 °C, and after 0.5 h and 4 h, aliquots were taken from the reaction mix and applied to the sucrose gradients. Centrifugation was performed at 22 °C for 3 h at 217,874 × *g* using an SW40 Ti rotor (Beckman Coulter). Fractions of 600 μl were carefully collected manually. During the manual fractionation, a thin film of low-density sucrose always remained on top of the gradients, which was then collected with the pellet fraction. Therefore, the protein amount in the last fraction is slightly overestimated. The pellet was resuspended in equal volumes of 1× Laemmli in PBS. Samples were analyzed by SDS-PAGE and immunoblotting. The amount of Tau in each fraction as a percentage of total Tau across the whole gradient was quantified using Image Studio Lite software (LI-COR Biosciences).

### SDS-PAGE and immunoblotting

Samples were run on either 4–20% Express Plus PAGE in Tris-MOPS-SDS running buffer (GenScript), 10% Criterion TGX gels (Bio-Rad) in Tris-glycine-SDS running buffer, or 3–8% Criterion XT Tris acetate gels (Bio-Rad) in Tris acetate-SDS running buffer. Proteins were transferred to PVDF membranes (Trans-Blot Turbo RTA transfer kit, Bio-Rad) using the Trans-Blot Turbo transfer system (Bio-Rad) and immunoblotted with the Tau antibody A-10 (1:1000–10,000, mouse, sc-390476, Santa Cruz Biotechnology) or anti-GFP (1:10,000, mouse, MMS-118P, Covance). An alkaline phosphatase-coupled secondary antibody (Vector Laboratories) together with ECF substrate (GE Healthcare Life Sciences) was used for development. The blots were imaged on an ImageQuant LAS-4000 (FUJIFILM Co.). Densitometric quantification of the signals was performed with Image Studio Lite software (LI-COR Biosciences).

### Dot blot

After centrifugation, the supernatant fractions were supplemented with 1% Sarkosyl and the pellets were resuspended in equal volumes of 1% Sarkosyl in disaggregation buffer by sonication. The samples were blotted onto a 0.2-μm nitrocellulose membrane (Roti-NC transfer membrane, Carl Roth) using the Bio-Dot apparatus (Bio-Rad). Subsequently, the membrane was immunoblotted with the Tau antibody HT7 (1:1000, mouse, MN1000, Thermo Fischer Scientific) and further processed as described above.

### Negative-stain EM

Tau fibrils alone or treated with chaperones in the absence or presence of ATP and an ATP-regenerating system were diluted in PBS or disaggregation buffer, respectively, and pipetted onto carbon-coated copper grids (Plano GmbH). Samples were allowed to absorb for 1 min before washing twice with 10 μl water for 1 min. Negative stain was achieved by incubation with 2% (w/v) aqueous uranyl acetate for 1 min. Excess solution was removed by blotting the grids carefully on filter paper before imaging on an EM-900 or an EM-910 electron microscope (Zeiss) with an accelerating voltage of 80 kV.

### Cell culture

The HEK293 cell line expressing 0N4R TauP301S-Venus, generated by McEwan *et al*. (23), was cultured in DMEM, high glucose, GlutaMAX supplement, pyruvate (Gibco) supplemented with 10% FCS (Gibco), 100 IU/ml penicillin, and 100 mg/ml streptomycin (Gibco) at 37 °C and 5% CO_2_. Cells were regularly tested for mycoplasma contamination.

### Generation of enriched seeded pool of TauP301S-Venus HEK293 cells

The naïve TauP301S-Venus HEK293 cell line was treated with preformed 1N4R Tau fibrils as published previously (23). Briefly, cells were plated in a 6-well plate in Opti-MEM reduced serum medium and GlutaMAX supplement (Gibco). The next day, the cells were treated with 100 nm preformed Tau fibrils and 10 μl Lipofectamine 2000 (Invitrogen) diluted in Opti-MEM. After 1 h of incubation time, equal volumes of complete DMEM were added. 3 days later, the cells were washed and resuspended in cell sorting buffer (1× PBS plus 0.8 mm EDTA, 0.5% [v/v] FCS) before carefully passing the cells through a 35-μm cell strainer (Corning). Focus-containing cells were sorted based on the intracellular distribution of Venus fluorescence (concentrated intensity signal *versus* diffuse distribution) on a FACS Aria IIIu (Becton Dickinson) with a 530/30-nm filter at the ZMBH FACS facility. Using these stringent sorting conditions, 7500 cells (2.5% of the original cell population), which contained a highly concentrated rather than a diffuse intracellular Venus signal, were collected in complete DMEM, expanded, and frozen. The presence of TauP301S-Venus foci before and after freezing/thawing was confirmed by fluorescence microscopy.

### Extraction of Sarkosyl-insoluble material from seeded TauP301S-Venus HEK293 cells

A confluent 15-cm dish of HEK293 cells propagating aggregated TauP301S-Venus was harvested by snap freezing. The cell pellet was resuspended in 400 μl cold extraction buffer (10 mm Tris, pH 7.5, 2 mm NaV, 50 mm NaF, 50 mm β-glycerophosphate, PhosSTOP phosphatase inhibitor [Roche], cOmplete EDTA-free protease inhibitor mixture [Roche], 100 mm NaCl) and sonicated briefly. Cell debris was removed by centrifugation at 1000 × *g*, 4 °C, for 1 min. The cleared lysate was centrifuged at 337,000 × *g*, 4 °C, for 15 min. The resulting pellet was resuspended in 100 μl extraction buffer with 1% (w/v) Sarkosyl, sonicated again, and then incubated for 1 h at 22 °C, 700 rpm, to extract the Sarkosyl-soluble fraction before repeating the centrifugation step. The Sarkosyl-insoluble pellet was resuspended in disaggregation buffer with protease inhibitors (50 mm Hepes-KOH [pH 7.5], 50 mm KCl, 5 mm MgCl_2_, 2 mm DTT, 2 mm NaV, 50 mm NaF, 50 mm β-glycerophosphate, PhosSTOP phosphatase inhibitor [Roche], cOmplete EDTA-free protease inhibitor mixture [Roche]), briefly sonicated, and centrifuged to remove the remaining detergent. Finally, the pellet was resuspended in disaggregation buffer with protease inhibitors and stored at 4 °C.

### Cell culture seeding assay

To test the seeding capacity of Tau liberated by the disaggregation machinery, disaggregation reactions were performed as described above. To obtain the fraction of Tau that was liberated by chaperone action, differential centrifugation steps first at 20,000 × *g* and then at 337,000 × *g* were performed, both for 30 min, at 4 °C. After ultracentrifugation, only the upper two-thirds of the supernatant were carefully collected, thereby avoiding disturbing the pelleted material. Samples of all fractions were subjected to SDS-PAGE and immunoblotting to confirm successful differential centrifugation.

The seeding assay with the biosensor TauP301S-Venus HEK293 was performed by following the protocol for liposome-mediated transduction by McEwan *et al*. (23). Briefly, 50,000 cells were seeded per 24 wells on poly-L-lysine-coated coverslips in 300 μl Opti-MEM reduced serum medium with GlutaMAX supplement (Gibco). The next day, the cells were treated with 25 μl of the 337,000 × *g* supernatants of the disaggregation reactions. The samples were mixed with 2.5 μl Lipofectamine 2000 (Invitrogen) in 200 μl OptiMEM and added to the cells. After 1 h of treatment, the seeding reaction was stopped by adding 500 μl complete DMEM to each well. 24 h later, the cells were fixed in 4% PFA in PBS for 30 min. After washing, the cells were incubated with 0.1 μg/ml DAPI in PBS and mounted in Vectashield (Vector Laboratories) for fluorescence microscopy.

To monitor the propagation of TauP301S-Venus foci over time, the cells were passaged for 27 days (6 passages) after seeding and imaged regularly with a Leica DM IL LED system equipped with a HI PLAN I Phase 2 40×/0.50 (Leica) objective lens.

### Microscopy and image analysis of fixed cells

Fixed TauP301S-Venus HEK293 cells were imaged using a Zeiss Cell Observer equipped with a Plan-Apochromat 20×/0.8 M27 (Zeiss) objective lens. For each image, three z-stacks at intervals of 4 μm were acquired to capture all foci within a cell. Semiautomated image analysis was performed using Fiji (57), and a macro for counting nuclei and for focus identification was developed together with the DKFZ Light Microscopy Core Facility. The foci then were manually assigned to single cells to calculate the percentage of cells in which Tau aggregation was seeded. Per replicate and condition, at least 288 cells were analyzed.

### Statistical analysis

The statistical analysis was performed using GraphPad Prism (GraphPad Software, version 6). For each data set, the sample size (*n*), *p* values, and statistical test applied are indicated in the corresponding figure legend.

All data are shown with individual data points and mean values ± S.D. with the following significance levels: nonsignificant (ns), *p* > 0.05; *, *p* ≤ 0.05; **, *p* ≤ 0.01; and ***, *p* ≤ 0.001.

## Data availability

All data are contained within the manuscript.

## Supplementary Material

Supporting Information
