# Supplementary material for: Disassembly of Tau fibrils by the human Hsp70 disaggregation machinery generates small seeding-competent species
Source: J Biol Chem. 2020 May 28;295(28):9676–90. doi: 10.1074/jbc.RA120.013478 (PMC7363153; doi:10.1074/jbc.RA120.013478)
Supplement: Supporting Information [file supp_295_28_9676__index.html]

Disassembly of Tau fibrils by the human Hsp70 disaggregation machinery generates small seeding-competent species — Amyloid Tau solubilization by molecular chaperones — Disassembly of Tau fibrils by the human Hsp70 disaggregation machinery generates small seeding-competent species — Amyloid Tau solubilization by molecular chaperones — Supporting Information 

# Disassembly of Tau fibrils by the human Hsp70 disaggregation machinery generates small seeding-competent species

## Supporting Information

- Figure 1D\_Source Data - Figure 1D\_Source Data
- Figure 1F\_Source Data - Figure 1F\_Source Data
- Figure 2C\_Source Data - Figure 2C\_Source Data
- Figure 3C\_Source Data - Figure 3C\_Source Data
- Figure 4C\_Source Data - Figure 4C\_Source Data
- Figure 5A\_Source Data - Figure 5A\_Source Data
- Figure 5B\_Source Data - Figure 5B\_Source Data
- Figure 5DE\_Source Data - Figure 5DE\_Source Data
- Figure 6E\_Source Data - Figure 6E\_Source Data
- Figures S1-S6 combined - Figures S1-S6 combined
