# Supplementary material for: Disassembly of Tau fibrils by the human Hsp70 disaggregation machinery generates small seeding-competent species
Source: J Biol Chem. 2020 May 28;295(28):9676–90. doi: 10.1074/jbc.RA120.013478 (PMC7363153; doi:10.1074/jbc.RA120.013478)
Supplement: Supporting Information [file supp_RA120.013478_159530_2_supp_531995_q5hmsr.pdf]

Supporting information

Figure S1

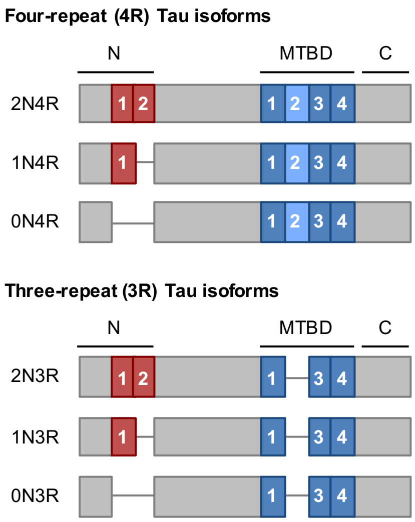

Figure S1. Alternative splicing of the human MAPT gene gives rise to six different Tau isoforms. The different isoforms are named based on their domain structures (left). Alternative splicing events in the N-terminal region (N1 and N2, red), give rise to the three different variants 2N, 1N, and 0N. The microtubule-binding domain (MTBD) consists of either three (3R) or four (4R) repeat domains (blue) and forms the major part of the fibril core in Tau amyloid assemblies. Alternative splicing of exon 10 (R2, light blue) within the MTBD determines the R variant (3R or 4R). The C-terminal region (C) is identical in all six human Tau variants.

Figure S2

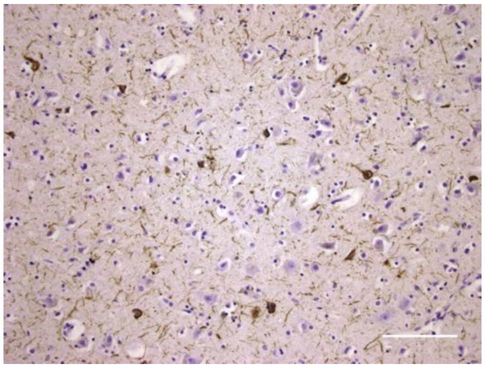

Figure S2. Tau pathology in the AD brain sample.
Light microscopy image of a section from the primary visual cortex of the brain sample used for extraction of sarkosyl-insoluble Tau filaments. The presence of numerous tangles in this brain area, as shown by phospho-Tau (AT8) staining, is indicative for Braak stage VI. Nuclei were counterstained with hematoxylin. Scale bar = 250 µm.

Figure S3

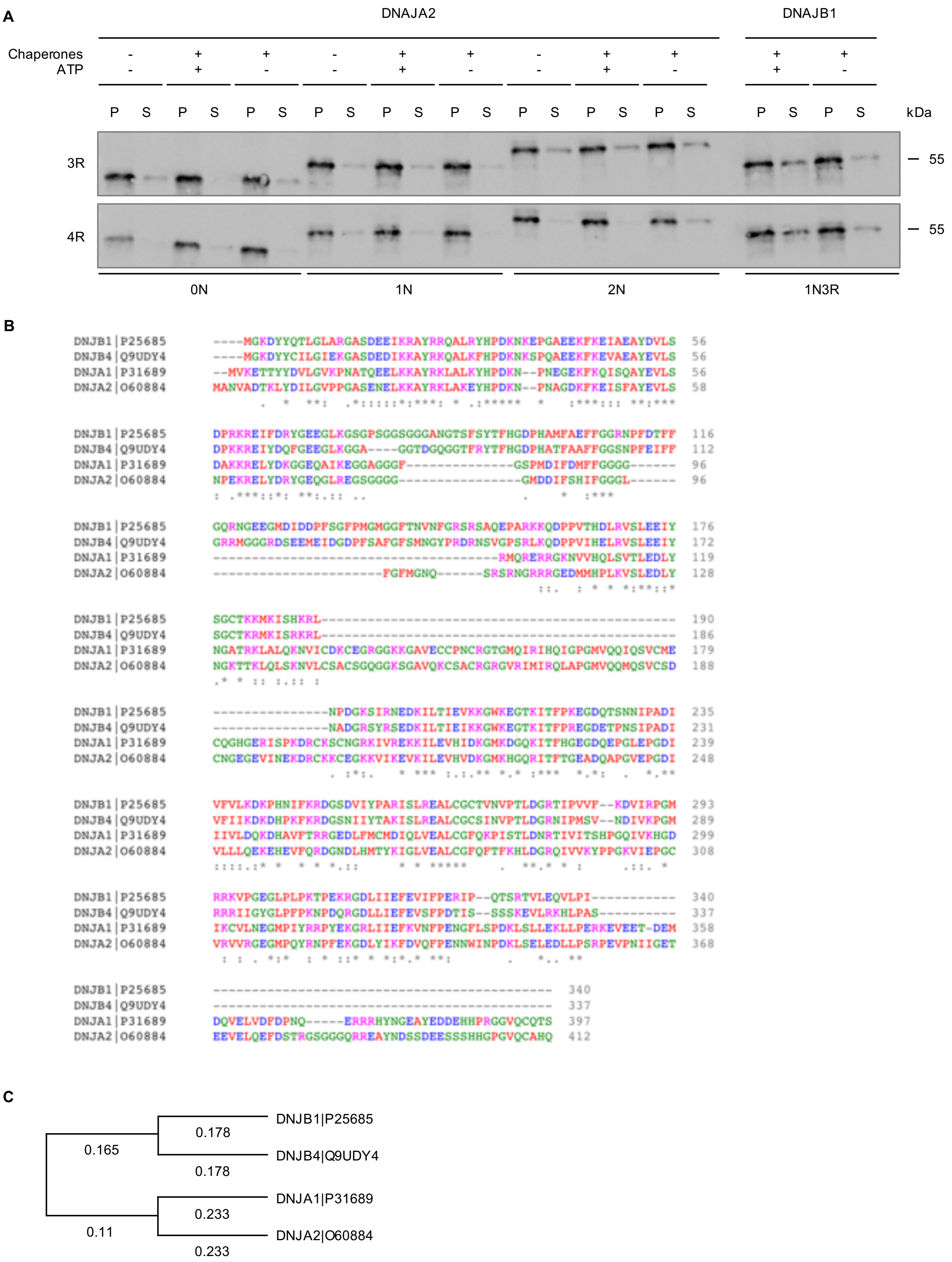
A
DNAJA2
DNAJB1
Chaperones
ATP
P S
kDa
3R
4R
55
0N
1N
2N
1N3R
B
DNJB1|P25685
DNJB4|Q9UDY4
DNJA1|P31689
DNJA2|O60884
C
DNJB1|P25685
DNJB4|Q9UDY4
DNJA1|P31689
DNJA2|O60884
0.178
0.178
0.165
0.233
0.233
0.11

Figure S3. Class B J-domain proteins mediate Tau disaggregation.
(A) Fibrils of all six Tau isoforms were treated with HSC70, HSPA4, and DNAJA2 in the presence or absence of ATP. S and P fractions were separated by centrifugation and Tau levels were analyzed by immunoblotting. DNAJA2 did not mediate Tau disaggregation *in vitro*. A disaggregation reaction with 1N3R fibrils and the canonical Hsp70 disaggregase (HSC70, DNAJB1, HSPA4) was included as a positive control on both blots to ensure correct exposure times.
(B) Sequence alignment of indicated J-domain proteins. Class B J-domain proteins DNAJB1 and DNAJB4 share a sequence identity of 65.7%.
(C) Phylogram of the class A and class B J-domain proteins analyzed in this study.

Figure S4

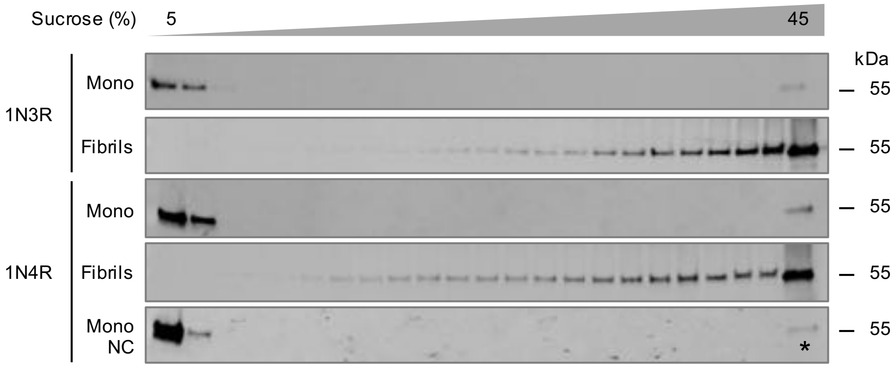

Figure S4. Characterization of monomeric and fibrillar Tau without the addition of chaperones. Monomeric Tau or fibrils were centrifuged over a 5-45% sucrose gradient. Fractions were collected manually, and the Tau content was analyzed by immunoblotting. Monomeric Tau was detected in the first two fractions, while Tau fibrils migrated to high-density fractions. Fractionating a gradient loaded with monomeric 1N4R Tau without previous centrifugation (no centrifugation, NC) demonstrated that minor amounts of monomeric Tau could be detected in the pellet fraction (marked with *) for technical reasons.

Figure S5

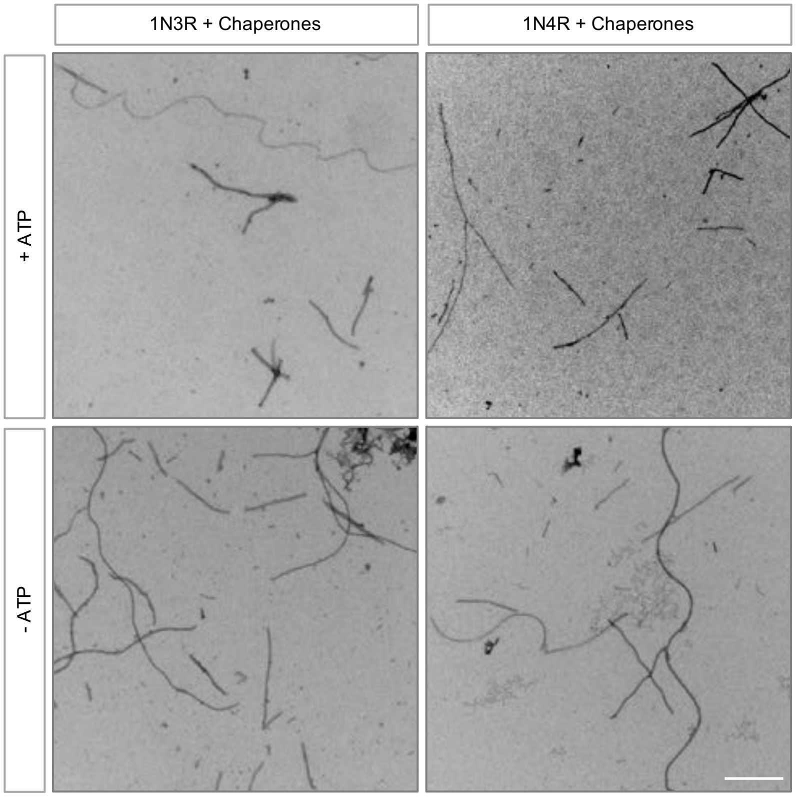

Figure S5. No accumulation of smaller Tau fragments after disaggregation.
Tau fibrils were treated for 20 h with the human Hsp70 disaggregation machinery in the presence or absence of ATP. Negative stained samples were analyzed by TEM. Scale bar = 500 nm.

Figure S6

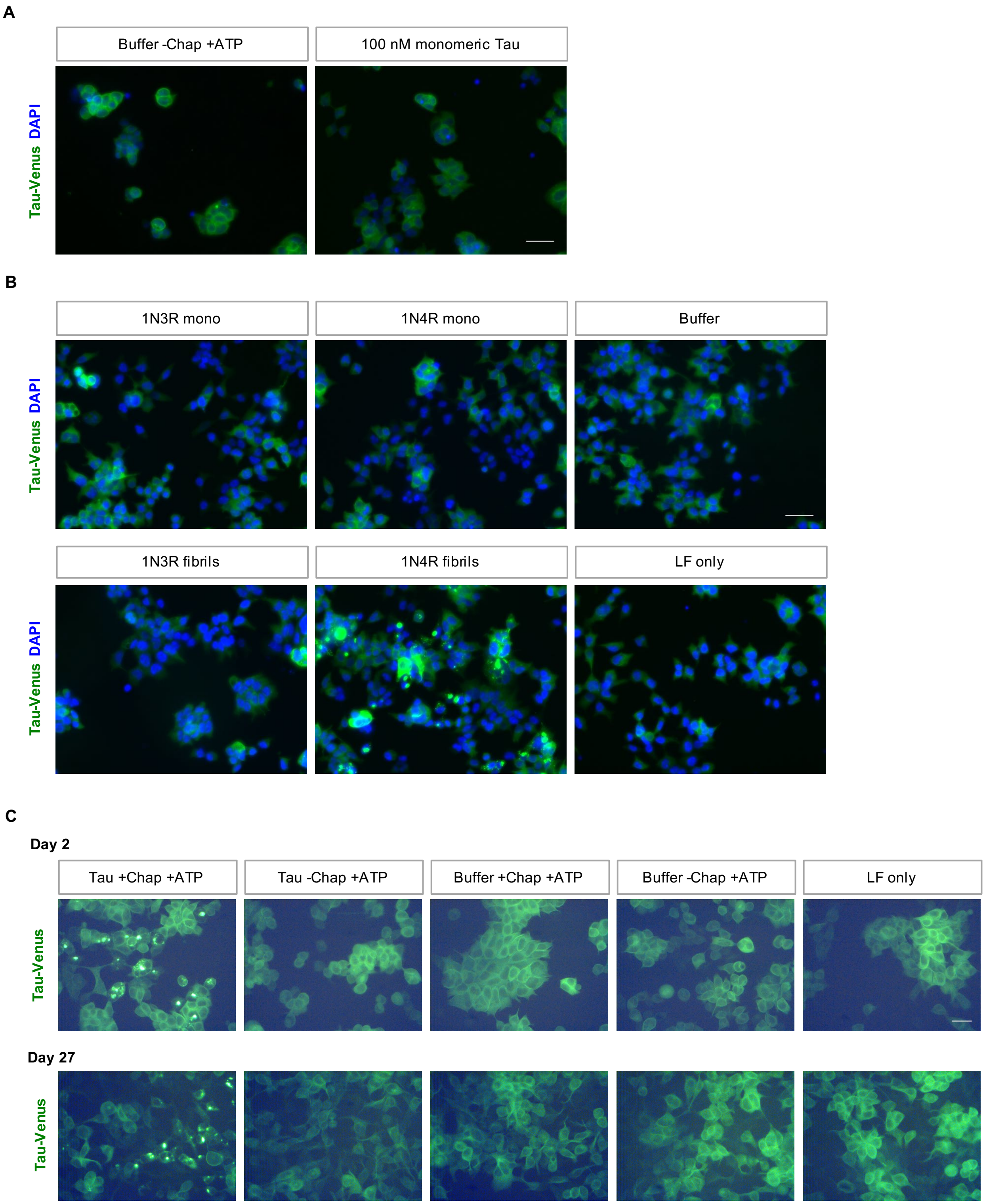
A
Buffer -Chap +ATP
100 nM monomeric Tau
Tau-Venus DAPI
B
1N3R mono
1N4R mono
Buffer
Tau-Venus DAPI
1N3R fibrils
1N4R fibrils
LF only
Tau-Venus DAPI
C
Day 2
Tau +Chap +ATP
Tau -Chap +ATP
Buffer +Chap +ATP
Buffer -Chap +ATP
LF only
Tau-Venus
Day 27
Tau-Venus

Figure S6. Tau disaggregation generates monomeric and small oligomeric seeding-competent species.
(A) Maximum intensity projections of fluorescence microscopy z-stacks of TauP301S-Venus HEK293 cells seeded with the 337 000 g supernatant fraction of the buffer control after the disaggregation reaction or naïve monomeric Tau. Neither caused foci formation in the TauP301S-Venus HEK293 cell culture model. The cells were fixed 24 h after the treatment. Scale bar = 50 µm.
(B) Maximum intensity projections of fluorescence microscopy z-stacks of TauP301S-Venus HEK293 cells seeded with 100 nM fibrils or monomer of 1N3R or 1N4R, respectively. Only fibrils assembled from 1N4R Tau seeded foci formation with high efficiency, but neither 1N3R fibrils, nor monomeric Tau of either isoform. Treatment with buffer or Lipofectamine in the absence of disaggregation buffer, chaperones, ATP, or fibrils (LF only) served as negative controls. Cells were fixed 48 h after the treatment. Scale bar = 50 µm.
(C) Epi fluorescence microscopy of TauP301S-Venus HEK293 cells treated with the 337 000 g supernatant fractions after the disaggregation reaction. Living cells were imaged 2 days and 27 days after the treatment. Treatment with Lipofectamine (LF only) served as a negative control. Scale bar = 50 µm.
